# Trends in Mushroom Exposures Reported to the Florida Poison Information Center Network, 2014–2024

**DOI:** 10.5811/westjem.56978

**Published:** 2026-10-14

**Authors:** Steven Chen, Rebecca Lipscomb, Rahul Mhaskar, Diana Dean

**Affiliations:** * University of South Florida Morsani College of Medicine, Tampa, Florida; † University of South Florida Morsani College of Medicine, Department of Medical Education, Tampa, Florida; ‡ University of South Florida Morsani College of Medicine, Department of Internal Medicine, Tampa, Florida; § University of South Florida, Department of Emergency Medicine, Tampa, Florida; || Florida Poison Information Center – Tampa, Tampa, Florida

## Abstract

**Introduction:**

Toxic mushroom exposures can occur through multiple mechanisms, including consumption as a delicacy, foraging, and use for psychoactive properties. Recent evidence suggests that psylocibin mushroom use is increasing in the United States. However, information on trends in mushroom poisonings in Florida is limited. This study aims to characterize trends in psychoactive and nonpsychoactive mushroom exposures reported to the Florida Poison Information Center Network (FPICN) over an 11-year period.

**Methods:**

This is a retrospective review of all mushroom exposures reported to the FPICN between 2014 to 2024. Our primary outcome was to evaluate the annual trend of psychoactive and nonpsychoactive exposures. Secondary outcomes included identifying patient demographics, exposure intent, clinical effects, and medical outcomes associated with each exposure type. Psychoactive mushrooms were defined as belonging to groups 2 (muscimol), 4 (muscarine and histamine), and 6 (psilocybin and psilocin) in the National Poison Data System coding manual. We performed statistical analyses using chi-square and Mann–Whitney tests, and joinpoint regression models.

**Results:**

A total of 2,995 mushroom exposures were reported. Psychoactive mushrooms comprised 15.6% (*n* = 467) of exposures and were more likely to involve males (72.5%, *P* < .001) and intentional ingestions (80.9%, *P* < .001). Joinpoint regression analysis showed that from 2014 to 2018, the rate of psychoactive mushroom exposures did not change significantly (annual percentage change [APC] = −1.5%; 95% CI, −35.9% to 19.3%; *P* = .88). In contrast, from 2018 through 2024, exposure rates increased significantly by an average of 34.4% per year (APC = 34.4%; 95% CI, 27.0%–60.4%, *P* < .001). These trends were standardized to overall poison center call volume. There were three deaths: a 67-year-old male (intentional–suspected suicide); an 81-year-old male (unintentional); and a 44-year-old female (unintentional), all involving mushrooms of unknown category.

**Conclusion:**

Psychoactive mushroom exposures in Florida have increased substantially since 2018, particularly among adolescents and adults, and were more commonly associated with intentional use. These findings may reflect changing cultural attitudes and increased accessibility. Further research is needed to better understand the evolving landscape associated with psychoactive mushroom use.

## INTRODUCTION

Mushroom poisoning is the ingestion of toxic substances produced by certain species of fungi, causing a wide range of clinical effects, from mild gastrointestinal distress to neuropsychiatric effects to severe end-organ damage.^1^ Most mushroom exposures are nonfatal and cause only minor effects.^2^ Most fatal poisonings originate from the cyclopeptide mushroom group, with *Amanita phalloides* accounting for the most fatalities.^2^ The National Poison Data System (NPDS), maintained by America’s Poison Centers, characterizes mushroom exposures by primary toxin or main symptoms into nine categories, including cyclopeptides, orellanine, muscimol, monomethylhydrazine, muscarine and histamine, hallucinogens (psilocybin and psilocin), coprine, gastrointestinal irritants, miscellaneous nontoxic and potentially toxic, and unknown.^3^

Toxic mushroom exposures can occur by a variety of mechanisms. In many cultures, certain mushroom species are prized as edible delicacies, although recent research has questioned the safety of some of these species.^4^ Mushroom foraging is also a popular tradition that occurs worldwide and in the United States. Foragers are at risk of misidentification of toxic mushroom species, especially in regions with a diverse array of toxic and nontoxic species.^2^ *Amanita phalloides*, for instance, is commonly misidentified by foragers for other edible mushroom species.^5^ Additionally, poisoning may also result from intentional ingestions of mushrooms for their psychoactive properties. Two main categories of psychoactive mushrooms exist: anticholinergic delirium-inducing mushrooms such as *Amanita muscaria;* and psilocybin mushrooms containing the lysergic acid diethylamide-like substance psilocybin and psilocin.

Between 1999 and 2016, 7,428 cases of mushroom poisoning were reported per year to US poison centers.^2^ The majority (86%) of these exposures involved mushrooms of unknown types; however among known exposures, psychoactive mushrooms were identified as the most common group. Notably, recent evidence indicates that the prevalence of hallucinogen use including psilocybin mushroom ingestions has been increasing in the US in the last decade.^6^,^7^ Although these studies have characterized mushroom exposures nationally, regional analysis in a state such as Florida, which has a unique climate, biodiversity, and population, remains limited.^6^

With the growing popularity of intentional psychoactive mushroom use, the goal of this study was to describe temporal trends, exposure characteristics, and clinical outcomes of psychoactive and nonpsychoactive mushroom exposures reported to Florida poison centers over an 11-year period.

## METHODS

This is a retrospective chart review of all mushroom exposures reported to the Florida Poison Information Center Network (FPICN), which includes exposures reported from home and/or healthcare institutions. The FPICN includes three poison centers across the state and, as a network, manages an average of 98,006 overall exposures per year. We obtained exposure data from the FPICN database from January 1, 2014 to December 31, 2024. Each record represents a single exposure or case, including the initial poison center call and any associated follow-up calls for that case. Records contain standardized codes derived from the National Poison Data System manual and entered by trained poison center personnel. No additional manual chart review was performed. Basic data quality checks were conducted to assess completeness and consistency of key variables. Records included call date, age, sex, reason for exposure, category of mushroom exposure, clinical effects, and medical outcomes. Medical outcome severities and categories of mushrooms were derived from the National Poison Data System coding manual (Appendix A).^3^ For this study, “psychoactive” mushroom exposures were defined by having the potential to cause mind-altering or neurobehavioral clinical effects. Psychoactive mushrooms were defined as those belonging to groups 2, 4, and 6. Figure 1 contains a study flow diagram illustrating excluded cases and the division between psychoactive and nonpsychoactive exposures.

**Population Health Research Capsule**

What do we already know about this issue?

*National data suggest that hallucinogen use, including psilocybin mushroom exposures reported to poison centers, has increased over the past decade.*

What was the research question?

*How have psychoactive and nonpsychoactive mushroom exposures changed over time in Florida?*

What was the major finding of the study?

*Since 2018, psychoactive mushroom exposures increased by 34.4% per year (95% CI, 27.0%–60.4; P < .001).*

How does this improve population health?

*Understanding the changing trends in psychoactive mushroom exposures in Florida can inform prevention efforts and poison center preparedness.*

We excluded records that were coded as informational requests on mushrooms. Exposures coded via routes that were not ingestions were also excluded, as well as exposures in which there were multiple identified generic mushroom categories.

Chi square tests were used to compare categorical independent variables between psychoactive and nonpsychoactive mushroom exposures. We used Mann–Whitney tests to compare patient age between psychoactive and nonpsychoactive exposures and joinpoint regression models to evaluate temporal trends. *P* values < .05 were considered statistically significant. Missing data for a specific variable (eg, age, sex, intent) were excluded from any analyses involving those variables. Analysis was done using SPSS, version 30 (IBM Corporation, Armonk, NY). The study was reviewed by the institutional review board (IRB) and deemed exempt by the host institution. Our study was compliant with the following medical record review criteria: case selection criteria; variable definition; abstraction forms; sampling method; and IRB approval.^8^ This study was conducted and reported in accordance with the Strengthening the Reporting of Observational Studies in Epidemiology (STROBE) guidelines for observational studies.

## RESULTS

From January 1, 2014, to December 31, 2024, 2,995 mushroom exposures were reported to the FPICN. The age distribution of all exposures ranged from 1 month to 96 years old, with a median age of 10. Exposures were most commonly unintentional (1,754/2,995 [58.6%]) and resulted in no effect (947/2995 [31.6%]). Throughout the 11-year study period, there were three reported deaths: a 67-year-old male with an unknown category mushroom exposure categorized as intentional, suspected suicide; an 81-year-old male with an unintentional, unknown category mushroom exposure; and a 44-year-old female with an unknown category mushroom exposure categorized as unintentional, misuse.

Most exposures (2,297/2,995; 76.7%) were coded as an unknown mushroom exposure. A total of 467 exposures (15.6%) were characterized as exposures to a psychoactive mushroom group (NPDS-defined groups 2, 4, 6). Specifically, muscarine and histamine mushrooms (group 4) were reported in 6 cases, muscimol (group 2) in 24 cases, and hallucinogenic varieties (psilocybin and psilocin, group 6) in 437 cases. Table 1 shows the number of exposures over the 11-year period by toxin group. Of the 467 exposures caused by psychoactive mushrooms, 378 (80.9%) were intentional, with most cases classified as abuse (298/467; 63.8%) followed by suspected suicides (60/467; 12.8%). Suspected suicides included suicide attempts and one completed suicide (the 67-year-old male referenced above).

Compared to nonpsychoactive exposures, psychoactive exposures were more likely to involve male patients (337/465 [72.5%] vs 1,538/2,520 [61.0%]) and intentional (378/467 [80.9%] vs 769/2,528 [30.4%]). Psychoactive cases were also more frequent among individuals aged 18 years and older (292/450 [64.9%] vs 833/2,461 [33.8%]) and those aged 6 to 17 years (124/450 [27.6%] vs 339/2,461 [13.8%]; all *P* < .001). Furthermore, psychoactive exposures resulted in higher rates of major (17/467 [3.6%] vs 34/2,528 [1.3%]), moderate (152/467 [32.5%] vs 235/2,528 [9.3%]), and minor medical exposures (178/467 [38.1%] vs 577/2,528 [22.8%]; all *P <* .001). Among psychoactive exposures, the most common clinical effects were hallucinations or delusions (149/467 [31.9%]), agitation (140/467 [30.0%]), tachycardia (104/467 [22.2%]), central nervous system depression (78/467 [16.7%]), confusion (71/467 [15.2%]), hypertension (64/467 [13.7%]), and vomiting (48/467 [10.3%]).

Among nonpsychoactive exposures, the most common clinical effects were vomiting (563/2,528 [22.3%]), nausea (380/2,528 [15.0%]), diarrhea (227/2,528 [9.0%]), abdominal pain (226/2,528 [8.9%]), tachycardia (140/2,528 [5.5%]), hallucinations or delusions (128/2,528 [5.1%]), agitation (120/2,528 [4.7%]), and hypertension (85/2,528 [3.4%]). Comparative statistics between psychoactive and nonpsychoactive exposures are shown in Table 2.

For psychoactive exposures between 2014 to 2024, joinpoint regression analysis identified one significant change in trend in 2018 (95% CI, 2016–2022). From 2014 to 2018, the rate of psychoactive mushroom exposures did not change significantly (APC = −1.5%; 95% CI, −35.9% to 19.3%, *P* = .88). In contrast, from 2018 through 2024, exposure rates increased significantly by an average of 34.4% per year (APC = 34.4%; 95% CI, 27.0%–60.4%, *P* < .001) (Figure 2). These temporal trends were standardized with the total call volume to FPICN each year between 2014 and 2024.

The increase in psychoactive mushroom exposures was observed across multiple age groups and exposure intents. The age groups of individuals 6 to 17 years of age and those 18 years or older had the largest absolute increase in psychoactive exposures over the study period. Among children younger than 6 years of age, there were no psychoactive exposures before 2019, increasing up to nine psychoactive exposures in 2022, 10 exposures in 2023, and nine exposures in 2024. When subcategorized by intent, unintentional psychoactive exposures increased from one or zero exposures per year between 2014 and 2018 to 17 exposures per year in both 2023 and 2024. Intentional psychoactive exposures increased from 12 exposures in 2014 to 91 exposures in 2024. The trend of psychoactive exposures categorized by intent and age group can be seen in Figure 3.

## DISCUSSION

This retrospective study provides insight into the changing landscape of mushroom exposures, especially psychoactive mushroom exposures, in Florida over the past 11 years. Our findings demonstrate an increase in psychoactive mushroom exposures, particularly after 2018, associated with notable rises in both intentional and unintentional exposures across multiple age groups. Our results align with broader societal trends, such as increased media coverage, public interest, and law enforcement seizures of psychoactive mushrooms.^9^–^11^ Our data also corroborate recent literature showing similar trends showing a significant increase in the number of psilocybin-related exposures in the last decade.^6^

Among all mushroom exposures, the majority were reported in children younger than six years of age, likely representing exploratory and unintentional ingestions common in this age group.^12^ In contrast, psychoactive mushroom exposures were significantly more common among children older than six, adolescents, and adults. These exposures were more likely to be intentional and were also associated with a higher incidence of major, moderate, and minor medical outcomes. Fatalities were rare and were all unknown mushroom exposures. It is unclear why psychoactive mushroom exposures were associated with a higher frequency of more severe medical outcomes. This result may be confounded by age, exposure intent, healthcare use, or other factors not evaluated in this study. Nevertheless, recent literature has demonstrated that psychoactive mushroom exposures can lead to a range of clinical effects, including mental status changes, dysrhythmias, and seizures.^13^,^14^

The rise in psychoactive mushroom exposures may reflect increased access to these substances as well as changing social perceptions concerning their use, potentially associated with decriminalization efforts and increased research and coverage of their therapeutic benefits.^15^–^17^ These changes may also reflect shifts in substance use patterns related to the stress and psychosocial effects of the COVID-19 pandemic, given that prior literature has demonstrated a rise in the use of other psychoactive substances, such as alcohol and cannabis, during the pandemic.^18^ The largest increase was observed in individuals 6 to 18 years of age, which includes adolescent exposures and is particularly concerning as it may suggest a need for targeted prevention efforts, especially in the context of social media influence and misperceptions about the safety of psilocybin-containing mushrooms. Increased exposures in younger children could reflect increased presence of psychoactive mushrooms in their home environment given known exploratory behaviors of this age group.^12^

## LIMITATIONS

The study had several notable limitations. Our dataset is subject to reporting bias because it may not be a complete representation of all cases of mushroom exposures in the FPICN region, given that this data relies on passive and voluntary reports from healthcare clinicians or calls from the public. Therefore, the number of true mushroom exposures in Florida was likely higher than the number captured in the FPICN database. Data may be skewed toward cases of higher complexity as clinicians may be less likely to report cases where medical management is considered relatively uncomplicated. Similarly, some hospitals with on-site toxicology services may manage mushroom exposures without consultation with the FPICN except in more severe cases.

Another significant limitation is the inability to confirm the identification or categorization of mushrooms for most exposure cases. The FPICN is a network of three different poison centers with differing staffing procedures and practices that may inherently vary. As a general practice, we encourage our specialists in poison information to obtain photographs of mushrooms whenever possible and discuss case details with the toxicologist on call. However, we were unable to extract data on which cases included photographs or what the identification result was. None of the FPICN toxicologists are mycologists, and while we are aware that consultation with a local mycologist may occur, we have no record of the cases on which they may have been consulted. Thus, it is not surprising that in most cases the mushrooms were unidentified (76%), which significantly limits the ability to report on exposure trends.

Additionally, data on the severity of medical outcomes were coded by NPDS coding definitions and was not independently clinically validated. They may therefore be influenced by reporter information and follow-up completeness. The temporal trends observed in this study may have been influenced by changes in FPICN coding practices, staffing, poison center awareness and usage, or reporting practices over time. Although our joinpoint analysis identified 2018 as an inflection point in the temporal trends, this was an exploratory finding rather than a prespecified outcome; therefore, these results should be interpreted with caution.

## CONCLUSION

Psychoactive mushroom exposures reported to Florida poison centers increased significantly during the study period, particularly intentional exposures among individuals older than six years of age. These trends may reflect cultural shifts, accessibility, changing attitudes toward hallucinogenic substances, or other factors not evaluated in this study. Further research should be conducted to better characterize the changing trends in mushroom exposures and their clinical implications.

## Supplementary Information



## Figures and Tables

**Figure 1:**
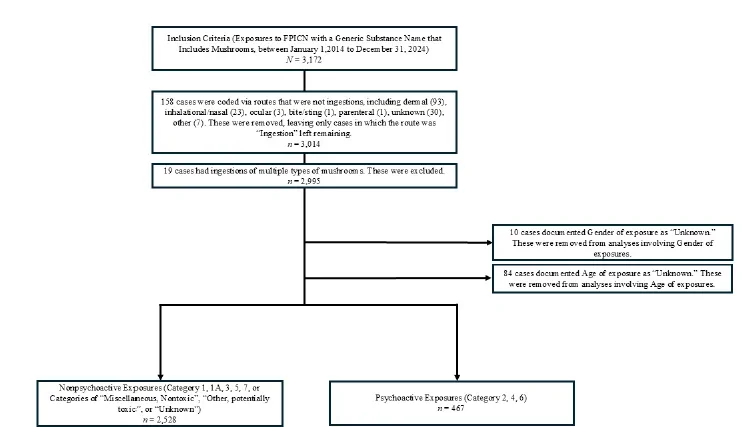
Study flow diagram for mushroom exposures reported to the Florida Poison Information Center Network between 2014–2024, sorted by presence or absence of psychoactive mushroom groups in each exposure. *FPICN*, Florida Poison Information Center Network.

**Figure 2:**
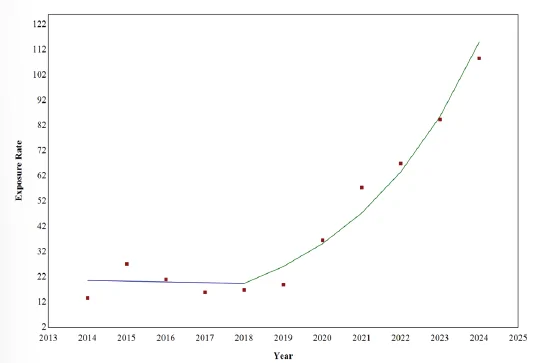
Joinpoint regression analysis of temporal trend of psychoactive mushroom exposures to Florida Information Center per year between 2014 and 2024. *Indicates that the Annual Percent Change is significantly different from zero at the alpha = .05 level. Final Selected Model: 1 Joinpoint. *APC*, annual percentage change.

**Figure 3:**
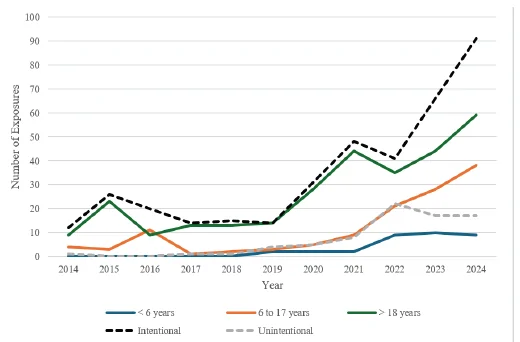
Psychoactive exposures per year reported to the Florida Poison Information Center by age group and intent.

**Table 1:** Mushroom exposures by toxin group in a study describing mushroom exposures reported to the Florida Poison Information Center.

1	Cyclopeptides	39
1A	Orellanine	1
2	Muscimol (Ibotenic Acid)*	24
3	Monomethylhydrazine (MMH)	0
4	Muscarine and histamine*	6
5	Coprine	1
6	Hallucinogenic (Psilocybin and Psilocin)*	437
7	Gastrointestinal irritants	95
	Miscellaneous, nontoxic, other potentially toxic	95
	Unknown	2,297

* Psychoactive mushrooms were defined as those belonging to groups 2, 4, and 6.

**Table 2:** Comparative statistics between psychoactive and nonpsychoactive mushroom exposures in a study describing mushroom exposures reported to the Florida Poison Information Center.

Variable		Nonpsychoactive (n = 2,528)	Psychoactive (n = 467)	Total (N = 2,995)	P value
Intentional/Unintentional	Intentional	769 (30.4)	378 (80.9)	1,147 (38.3)	< .001
	Unintentional	1,678 (66.4)	76 (16.3)	1,754 (58.6)	
	Other	50 (2)	3 (0.6)	53 (1.8)	
Sex**	Male	1,538 (61)	337 (72.5)	1,875 (62.8)	< .001
	Female	982 (39)	128 (27.5)	1,110 (37.2)	
Age group**	< 6 years	1,289 (52.4)	34 (7.6)	1,323 (45.4)	< .001
	6–17 years	339 (13.8)	124 (27.6)	463 (15.9)	
	> 18 years	833 (33.8)	292 (64.9)	1,125 (38.6)	
Medical outcome	Followed to known outcome—death or indirect report of death	3 (0.1)	0 (0)	3 (0.1)	< .001
	Followed to known outcome—major effect	34 (1.3)	17 (3.6)	51 (1.7)	
	Followed to known outcome— moderate effect	235 (9.3)	152 (32.5)	387 (12.9)	
	Followed to known outcome—minor effect	577 (22.8)	178 (38.1)	755 (25.2)	
	Followed to known outcome—no effect	923 (36.5)	24 (5.1)	947 (31.6)	
	Unable to follow—judged potentially toxic exposure	392 (15.5)	72 (15.4)	464 (15.5)	
	Not followed—judged non-toxic	75 (3)	1 (0.2)	76 (2.5)	
	Not followed—judged minimal clinical effects possible	289 (11.4)	23 (4.9)	312 (10.4)	
Age (years)*		5 [2,22]	20 [17,28]	10 [2,24]	< .001

Values are N(%) unless otherwise specified.

* Median.

** 10 cases were missing values for sex and removed from analysis involving sex; 84 cases were missing values for age and removed from analysis involving age.
