# Supplementary material for: Trends in Mushroom Exposures Reported to the Florida Poison Information Center Network, 2014–2024
Source: West J Emerg Med. 2026 Oct 14;27(6):1720–6. doi: 10.5811/westjem.56978 (PMC13761819; doi:10.5811/westjem.56978)
Supplement: Supplementary file 1 [file wjem-27-1720-s001.docx]

| Appendix A: Categories of Mushroom Intoxications and Medical Outcomes in a Study Describing Mushroom Exposures reported to Florida Poison Information Center |  |
| --- | --- |
|  | |
| Categories of Mushroom Intoxications: |  |
| 1 | Cyclopeptides |
| 1A | Orellanine |
| 2 | Muscimol (Ibotenic Acid) |
| 3 | Monomethylhydrazine (MMH) |
| 4 | Muscarine and Histamine |
| 5 | Coprine |
| 6 | Hallucinogenic (Psilocybin and Psilocin) |
| 7 | Gastrointestinal Irritants |
| Unk | Miscellaneous, nontoxic, other potentially toxic |
| Misc | Unknown |
|  | |
| Categories of Medical Outcomes: |  |
| No effect | The patient did not develop any signs or symptoms as a result of the exposure. |
| Minor effect | The patient developed some signs or symptoms as a result of the exposure |
| Moderate effect | The patient exhibited signs or symptoms as a result of the exposure that were more pronounced, more prolonged, or more systemic in nature than minor symptoms |
| Major effect | The patient exhibited signs or symptoms as a result of the exposure that were life-threatening or resulted in significant residual disability or disfigurement. |
| Death | The patient died as a result of the exposure or as a direct complication of the exposure. |
